# Ischemic Preconditioning Did Not Affect Central and Peripheral Factors of Performance Fatigability After Submaximal Isometric Exercise

**DOI:** 10.3389/fphys.2020.00371

**Published:** 2020-04-28

**Authors:** Martin Behrens, Volker Zschorlich, Thomas Mittlmeier, Sven Bruhn, Florian Husmann

**Affiliations:** ^1^Institute of Sport Science, University of Rostock, Rostock, Germany; ^2^Department of Traumatology, Hand and Reconstructive Surgery, University Medicine Rostock, Rostock, Germany

**Keywords:** central fatigue, peripheral fatigue, muscle fatigue, contractile function, effort perception, muscle pain, pain perception, perceived fatigability

## Abstract

The present study was designed to provide further insight into the mechanistic basis for the improved exercise tolerance following ischemic preconditioning (IPC) by investigating key-determinants of performance and perceived fatigability. Using a randomized, counterbalanced, single-blind, sham-controlled, crossover design, 16 males performed an isometric time-to-exhaustion test with the knee extensors at 20% maximal voluntary torque (MVT) after an IPC and a sham treatment (SHAM). Those who improved their time-to-exhaustion following IPC performed a time-matched IPC trial corresponding to the exercise duration of SHAM (IPC_tm_). Neuromuscular function was assessed before and after exercise termination during each condition (IPC, IPC_tm_, and SHAM) to analyze the impact of IPC on performance fatigability and its central and peripheral determinants. Muscle oxygenation (SmO_2_), muscle activity, and perceptual responses (effort and muscle pain) were recorded during exercise. Performance fatigability as well as its central and peripheral determinants were quantified as percentage pre-post changes in MVT (ΔMVT) as well as voluntary activation (ΔVA) and quadriceps twitch torque evoked by paired electrical stimuli at 100 and 10 Hz (ΔPS100 and ΔPS10⋅PS100^–1^ ratio), respectively. Time-to-exhaustion, performance fatigability, its determinants, muscle activity, SmO_2_, and perceptual responses during exercise were not different between IPC and SHAM. However, six participants improved their performance by >10% following IPC (299 ± 71 s) compared to SHAM (253 ± 66 s, *d* = 3.23). The time-matched comparisons (IPC_tm_ vs. SHAM) indicated that performance fatigability, its determinants, and SmO_2_ were not affected, while effort perception seemed to be lower (η_p_^2^ = 0.495) in those who improved their time-to-exhaustion. The longer time-to-exhaustion following IPC seemed to be associated with a lower effort perception (η_p_^2^ = 0.380) and larger impairments in neuromuscular function, i.e., larger ΔMVT, ΔVA, and ΔPS10⋅PS100^–1^ ratio (*d* = 0.71, 1.0, 0.92, respectively). IPC did neither affect exercise tolerance, performance fatigability, as well as its central and peripheral determinants, nor muscle activity, SmO_2_, and perceptual responses during submaximal isometric exercise. However, IPC seemed to have an ergogenic effect in a few subjects, which might have resulted from a lower effort perception during exercise. These findings support the assumption that there are ‘responders’ and ‘non-responders’ to IPC.

## Introduction

Ischemic preconditioning (IPC) involves repeated, short-term periods of vascular occlusion with subsequent reperfusion and has been shown to increase human performance if applied to the exercising limb prior to physical activity (Incognito et al., 2016). For example, studies have found that IPC has ergogenic effects on a variety of endurance exercise modalities including running (Bailey et al., 2012), cycling (de Groot et al., 2010), swimming (Ferreira et al., 2016), and sustained submaximal isometric contractions (Tanaka et al., 2016). However, besides the observation of an enhanced performance, a number of studies have also reported no or even detrimental effects of IPC on endurance performance (Incognito et al., 2016; Sabino-Carvalho et al., 2017; Marocolo et al., 2019). Although the exact physiological mechanisms underlying the performance-enhancing effect of IPC still need to be clarified, improved metabolic efficiency and/or blood flow in the active skeletal muscles (Incognito et al., 2016) as well as neural adjustments (Cruz et al., 2015; Marocolo et al., 2019) have been discussed. The physiological alterations associated with IPC are thought to delay fatigue development and thereby increase endurance performance (Tanaka et al., 2016; Cruz et al., 2017). In this context, fatigue can be defined as a psychophysiological symptom that is characterized by an impaired physical and/or cognitive function as a result of interactions between performance and perceived fatigability. Performance fatigability can be characterized as the decline of an objective performance measure over time. In the present study, performance fatigability refers to the exercise-induced impairment in maximal voluntary torque-generating capacity of the involved muscles (traditionally termed muscle fatigue) caused by a decrease in voluntary activation of muscles and/or alterations at or distal to the neuromuscular junction that result in contractile dysfunction (traditionally termed central and peripheral fatigue, respectively). Perceived fatigability refers to the perceptual milieu during fatiguing exercise that emerges from homeostatic challenges of different physiological systems and the psychological state of the individual. During ongoing physical activity, perceived fatigability is thought to affect the integrity of the performer and thereby contributes to the regulation of exercise behavior and ultimately motor performance (Kluger et al., 2013; Enoka and Duchateau, 2016; Venhorst et al., 2018).

Recently, Halley et al. (2018, 2019) have found that IPC does not alter central and peripheral determinants of performance fatigability after maximal voluntary isometric and isokinetic exercise of the knee extensors compared to a sham treatment (SHAM). However, maximal voluntary contractions (MVC) induce complete or near-complete ischemia (Oranchuk et al., 2019), which renders modulations of blood flow due to IPC unlikely. To the authors’ knowledge, there is no study to date that has investigated the impact of IPC on central and peripheral mechanisms of performance fatigability as well as its effect on determinants of perceived fatigability (i.e., perception of effort and exercise-induced muscle pain) during submaximal endurance exercise. Therefore, the present study was designed to provide further insight into the mechanistic basis for the improved exercise tolerance that has been shown following IPC. We have specifically chosen a time-to-exhaustion test that consisted of a sustained isometric knee extension at 20% maximal voluntary torque (MVT), because Tanaka et al. (2016) have found an improved exercise tolerance of 17.2% together with an accelerated muscle deoxygenation response during exercise after IPC using this protocol.

Based on the results of Tanaka et al. (2016), we hypothesized that performance fatigability, its determinants, and perceptual responses are affected by IPC compared to SHAM.

## Materials and Methods

### Participants

An *a priori* sample size calculation was conducted based on the effect size of a previously published study investigating the impact of IPC on time-to-exhaustion during a sustained isometric knee extension (Tanaka et al., 2016). A two-sided significance level of 0.05, a correlation between groups of 0.5, and a power of 0.95 indicated that 13 participants would be required. To account for potential drop out, 16 recreationally active male subjects were recruited to participate in the present study (age: 26 ± 4 years, height: 183 ± 6 cm, body weight: 81 ± 8 kg, systolic blood pressure: 132 ± 6 mmHg, diastolic blood pressure: 77 ± 6 mmHg, training hours per week: 9 ± 3 h). Based on the common finding that performance fatigability depends on sex (for a review see Hunter, 2014), a sample comprising exclusively male participants was chosen. Subjects were excluded if they were hypertensive (>140/90 mmHg) or had more than one risk factor for thromboembolism (Motykie et al., 2000). All participants were familiar with endurance exercise. Subjects were asked to abstain from vigorous exercise, analgesics, caffeine, alcohol, and nitrate-rich food consumption for 24 h prior to the laboratory visits. The study was approved by the university ethics committee and was conducted according to the declaration of Helsinki. All subjects were informed about possible risks and discomfort associated with the investigations prior to giving their written consent to participate.

### Experimental Procedure

All participants visited the laboratory on at least three different occasions. During the first visit, subjects’ arterial occlusion pressure as well as their MVT of the knee extensors were determined. Furthermore, participants were thoroughly familiarized with the following procedures: (i) neuromuscular tests comprising MVCs combined with peripheral nerve stimulation, (ii) the fatigue protocol (a sustained isometric knee extension at 20% MVT) as well as (iii) ratings of perceived effort and exercise-induced leg muscle pain.

**FIGURE 1:**
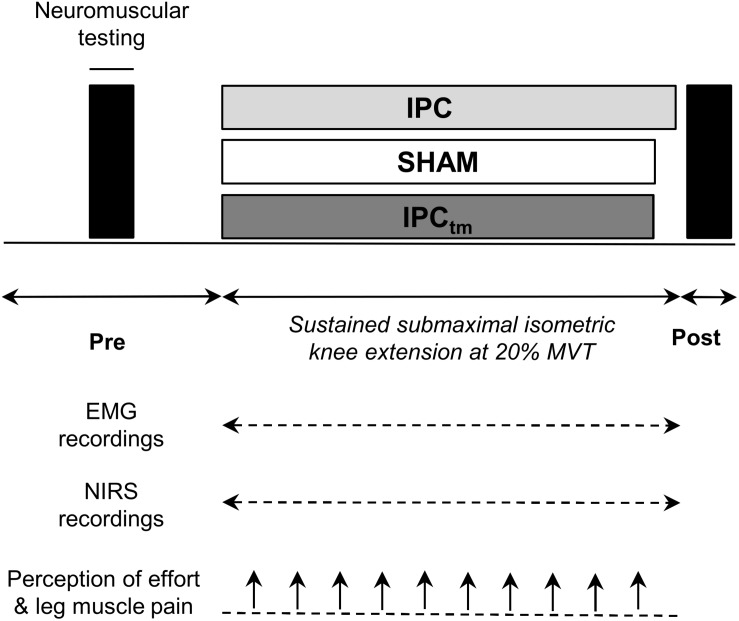
Schematic representation of the experimental design. Participants performed an isometric time-to-exhaustion test with the knee extensors at 20% maximal voluntary torque (MVT) following ischemic preconditioning (IPC) and a sham treatment (SHAM). Those who improved their time-to-exhaustion following IPC were considered as ‘responders’ and performed a time-matched IPC trial corresponding to the SHAM exercise duration (IPC_tm_). In case IPC has an ergogenic effect on time-to-exhaustion, this procedure allows a time-matched comparison of neuromuscular data between IPC and SHAM. Electromyography (EMG) and near-infrared spectroscopy (NIRS) data were continuously recorded during exercise. Effort perception and exercise-induced leg muscle pain were recorded every 30 s during exercise.

Using a randomized, counterbalanced, single-blind, sham-controlled, crossover design, participants performed a time-to-exhaustion test with a sustained unilateral isometric knee extension at 20% MVT following (i) an IPC protocol and (ii) a SHAM protocol, respectively. The experimental trials were separated by 7 ± 1 d. Participants who improved their time-to-exhaustion following IPC by at least 10% were considered as ‘responders’ and performed a time-matched IPC trial corresponding to the SHAM exercise duration (IPC_tm_). The cut-off value of 10% was chosen because studies have shown that the coefficient of variation for time-to-exhaustion is ∼8% for submaximal isometric and dynamic knee extensions (Zech et al., 2008; Pageaux et al., 2016). In case IPC has an ergogenic effect on time-to-exhaustion, this procedure allows a time-matched comparison of neuromuscular data between IPC and SHAM (Figure 1). All conditions (IPC, IPC_tm_, and SHAM) were further examined to analyze the impact of IPC on (i) performance fatigability, (ii) central and peripheral aspects of performance fatigability, (iii) muscle O2 saturation (SmO_2_), (iv) electromyographic (EMG) activity as well as (v) perception of effort and exercise-induced leg muscle pain. This experimental procedure was chosen based on the observation that some people seem to respond to IPC but others not (Incognito et al., 2016; Marocolo et al., 2019).

Prior to the baseline measurements, participants performed an initial warm-up on a stationary bicycle (5 min, 100 W, 90 rpm) followed by a specific warm-up on a dynamometer comprising two isometric contractions for 5 s at 50, 70, and 90% of MVT interspaced by 60 s of rest (MVT was determined during the familiarization session), respectively. Baseline neuromuscular tests were performed before the IPC and SHAM protocols to exclude any effects of the study interventions on these measures. Neuromuscular tests consisted of supramaximal electrical stimulations of the femoral nerve during and after isometric MVCs. Afterward, subjects performed two to three short sustained isometric voluntary contractions at 20% MVT to acutely familiarize them with the exercise protocol. Participants were again familiarized with the ratings of perceived effort and exercise-induced leg muscle pain. During the study interventions, i.e., IPC and SHAM, the principal investigator left the room to rule out instruction differences between conditions due to the awareness of the protocol. An additional warm-up on a stationary bicycle (5 min, 100 W, 90 rpm) was performed before the fatigue protocol. SmO_2_ as well as EMG activity were continuously recorded during each experimental condition. Every 30 s during the fatiguing protocol, participants were asked to rate their perceived effort and exercise-induced leg muscle pain. Neuromuscular tests were again performed immediately after (<10 s) exercise termination to investigate the development of performance fatigability.

All measurements were carried out on the quadriceps muscle of the dominant leg (i.e., kicking preference). During neuromuscular testing and the fatiguing protocol, subjects were comfortably seated and secured on a CYBEX NORM dynamometer (Computer Sports Medicine^®^, Inc., Stoughton, MA, United States). The seating position was adjusted for each participant and settings were documented for the subsequent sessions.

### Study Interventions

The IPC and SHAM protocols were applied to the participants in a supine position. Arterial occlusion pressure was determined as described in a previously published study from our laboratory (Husmann et al., 2018). Shortly, a pneumatic cuff (10 × 76 cm, Ulrich Medical, Ulm, Germany) was placed on the most proximal part of the thigh and was inflated using a cuff inflator system (Heidi^TM^; UlrichMedical, Ulm, Germany) until the pulse of the tibial artery, which was monitored using a handheld bidirectional Doppler probe (Hadeco Bidop ES-100V3, Kawasaki, Japan), was interrupted (arterial occlusion pressure: 210 ± 20 mmHg). During the IPC and SHAM protocol, the cuff was inflated to 120% of subjects’ arterial occlusion pressure (254 ± 24 mmHg) or 20 mmHg (SHAM) for three cycles of 5 min interspersed with 5 min of reperfusion. This protocol has been shown to increase exercise tolerance during a sustained isometric contraction at 20% of MVC in healthy young males by 17.2% (Tanaka et al., 2016). No adverse effects were observed or reported during these procedures. The time interval between the study interventions and the start of the fatiguing task was 20 min. None of the participants had previously used or had knowledge about IPC. Furthermore, they were instructed that both IPC and SHAM increase exercise performance and that the aim of the study is to identify the most effective protocol.

### Submaximal Fatigue Protocol

Exercise tolerance was quantified via the use of dynamometer-based single-joint endurance exercise, which provides a suitable model to investigate the underlying mechanisms of performance fatigability without a significant time delay for the assessment of neuromuscular function and cardiorespiratory limitations typically associated with whole-body endurance exercise. Therefore, the fatigue protocol comprised a sustained unilateral isometric knee extension at 20% MVT until exhaustion at 90° knee flexion (0° = full extension). The MVT recorded on the respective day served as the reference. This exercise protocol was chosen because it has been shown that time-to-exhaustion was improved during this task following IPC compared to a control condition (Tanaka et al., 2016). On each visit of the laboratory, subjects performed two to three short sustained isometric voluntary contractions at 20% MVT to acutely familiarize them with the exercise protocol. This was done after the neuromuscular baseline measurements and before the study interventions, i.e., IPC and SHAM. The participants were provided with visual feedback and had to match a target torque displayed on a digital oscilloscope (HM1508, HAMEG Instruments, Mainhausen, Germany). Exhaustion was defined as a decrease in torque by more than 10% for a duration of more than 5 s despite strong verbal encouragement by the principal investigator. Neither the participants nor the principal investigator who terminated the task were aware of the elapsed time. In order to motivate the participants to exercise for as long as possible during the time-to-exhaustion test, monetary rewards were announced for the three best performances (50 €, 30 €, 20 €). The subjects and the principal investigator did not get feedback about the performance until the completion of the study.

### Torque Recordings

Electrically evoked and voluntary torques were measured using a CYBEX NORM dynamometer (Computer Sports Medicine^®^, Inc., Stoughton, MA, United States). Participants were seated on an adjustable chair with the knee and hip fixed at 90° and 80° (0° = full extension), respectively. In order to avoid excessive movements of the participants during data recording, they were fixed with straps at the waist and chest. The subjects’ lower leg was affixed to the lever arm of the dynamometer and the dynamometer rotation axis was aligned with the knee joint rotation axis. During isometric strength testing, subjects were instructed to cross their arms in front of their chest and to push as hard as possible against the lever arm of the dynamometer. Strong verbal encouragement was given by the investigator and visual feedback of the torque-time curve was provided on a digital oscilloscope (HM1508, HAMEG Instruments, Mainhausen, Germany). Torque signals were digitized with a sampling frequency of 3 kHz using an analog-to-digital converter (NI PCI-6229; National Instruments, Austin). Data were saved on a hard drive for later analysis using a custom-built LABVIEW based program (Imago, Pfitec, Germany).

### Electrical Nerve Stimulation

Electrical femoral nerve stimulation was utilized to assess neuromuscular function of the quadriceps muscle. A constant-current stimulator (Digitimer DS7A, Herfordshire, United Kingdom) was used to deliver square-wave pulses of 1 ms duration with maximal voltage of 400 V. After determining the optimal site for electrical stimulation in the femoral triangle, the position was marked onto the participants’ skin to ensure repeatable measurements within each session. During neuromuscular testing, a ball probe cathode (15 mm diameter) was pressed into the femoral triangle always by the same experienced investigator to guarantee optimal electrical stimuli delivery. Individual stimulation intensity was progressively increased until the maximum compound muscle action potential (M_max_) of vastus medialis (VM), rectus femoris (RF), and vastus lateralis (VL) muscles as well as a plateau in knee extensor twitch torque was achieved. During the subsequent testing procedures, the stimulation intensity was increased by additional 40% to guarantee supramaximal stimulation. A self-adhesive electrode (35 × 45 mm, Spes Medica, Genova, Italy) served as the anode and was affixed over the greater trochanter. Potentiated quadriceps twitch torques evoked by paired electrical stimuli at 100 Hz (PS100), 10 Hz (PS10), and single stimuli (SS) were elicited 2, 4, and 6 s following the isometric MVCs, respectively. Voluntary activation of the quadriceps muscle during isometric MVCs was quantified using the interpolated twitch technique. Therefore, electrical paired stimuli (PS100) were automatically delivered to the femoral nerve 2 s after torque onset (during the plateau phase) and 2 s after the MVCs.

### EMG Recordings During Exercise

A detailed description of the EMG recordings can be found in a previously published study from our group (Behrens et al., 2015). Briefly summarized, myoelectrical signals of the VM, RF, and VL were recorded using surface electrodes (EMG Ambu Blue Sensor N). EMG signals were amplified (2500×), band-pass filtered (10–450 Hz), and digitized with a sampling frequency of 3 kHz using an analog-to-digital converter (NI PCI-6229, National Instruments, Austin, United States). Data were saved on a hard drive for later analysis using a custom-built LABVIEW based program (Imago, Pfitec, Germany).

### Muscle Oxygenation During Exercise

SmO_2_ reflects the balance between O_2_ delivery and O_2_ demand in the analyzed muscle (Ferrari et al., 2011). A portable near-infrared spectroscopy (NIRS) device (Moxy, Fortiori Design LLC, Minnesota, United States) was used to continuously monitor SmO_2_ of the VL. The Moxy monitor enables reliable measurements of SmO_2_ (Crum et al., 2017). The participants’ skin was shaved and cleaned prior to optode placement. The NIRS probe was attached at mid-thigh level, closely to the VL EMG electrodes, and was secured with tape and covered with a protective shell to avoid artifacts caused by motion and light. Reliable optode placement between sessions was ensured by documenting the distance to the patella, measured from the participants’ patella to the greater trochanter. Additionally, skinfold thickness above the VL was measured using a skinfold caliper (4 ± 1 mm). Signals were recorded with a sampling frequency of 2 Hz.

### Ratings of Perceived Effort and Exercise-Induced Leg Muscle Pain

The participants were briefed about how to rate perceived effort and exercise-induced leg muscle pain during the familiarization session as well as during the subsequent visits of the laboratory. The 15-point Borg scale (Borg, 1982) was used to quantify subjects’ perception of effort. The participants received written instructions based on recently proposed guidelines (Pageaux, 2016) during each testing session. The instructions comprised the definition of effort (“the conscious sensation of how hard, heavy, and strenuous a physical task is”), exercise-specific descriptions (“How hard is it for you to drive your leg?”), exercise-anchoring (e.g., “maximal exertion corresponds to the effort you experienced while you were performing a MVC”) and the distinction of effort, exercise-induced leg muscle pain, and other exercise-related sensations. Exercise-induced leg muscle pain was assessed using a modified category-ratio 10 (CR-10) scale (Cook et al., 1997). Leg muscle pain during exercise was defined as the perceived pain intensity exclusively in the exercising quadriceps muscle. The participants were asked to rate their perceived effort and exercise-induced leg muscle pain every 30 s during the fatigue protocol.

### State Fatigue

It has been shown that state fatigue induced by sustained cognitive activity can be detrimental to subsequent endurance performance (Marcora et al., 2009). Therefore, state fatigue was quantified before each testing session using the fatigue scale of the Profile of Mood States (POMS-F) (Behrens et al., 2018). The POMS-F has been shown to provide a reliable and valid instrument to assess the level of state fatigue across a wide range of cohorts (O’Connor, 2004). In case of a difference between conditions, state fatigue would have been considered as a covariate in the statistical analyses.

### Data Analyses

Time-to-exhaustion was defined as the time from the onset of exercise to task failure. Performance fatigability was quantified via the percentage change in MVT values from pre- to post-exercise (ΔMVT). Percentage changes in voluntary activation (ΔVA) and PS100 (ΔPS100) from pre- to post-exercise were used to quantify central and peripheral factors of performance fatigability, respectively. All torque signals were corrected for the effect of gravity. Isometric MVT was defined as the highest torque value prior to the electrically evoked superimposed twitch torque. Peak twitch torques (i.e., highest values of the torque-time curve) were determined for PS100, PS10, and SS, respectively. The PS10⋅PS100^–1^ torque ratio was calculated as an index of low-frequency fatigue and reduced values are thought to indicate impairments in excitation-contraction coupling (Verges et al., 2009). The level of voluntary activation was calculated using the corrected formula: [1 – superimposed twitch (T_b_ × MVT ^–1^) × control twitch^–1^] × 100 (Strojnik and Komi, 1998). MVT is the maximal torque level and T_b_ the torque value immediately before the electrically evoked superimposed twitch torque. The corrected formula is used to avoid the potential problem that the superimposed stimuli are not always applied during MVT. As shown recently by our group, voluntary activation of the knee extensors can be reliably assessed during isometric contractions using the corrected formula (Behrens et al., 2017).

M_max_ amplitudes elicited by electrical nerve stimulation were measured peak-to-peak. Muscle activity during exercise was assessed by calculating the root mean square of the EMG signal (RMS-EMG) averaged for 10 s at the beginning, as well as at 25, 50, 75, and 100% of the shortest trial (IPC or SHAM), respectively. Data of the other trial was calculated for the same points in time. The same was done for the IPC_tm_ condition.

RMS-EMG of VM, RF, and VL were normalized to their corresponding M_max_ values (RMS⋅M^–1^) and averaged to give an index of quadriceps muscle activation (Q RMS⋅M^–1^) (Husmann et al., 2019).

A 4th order low-pass zero-phase Butterworth filter (cutoff frequency 0.2 Hz) was applied to the NIRS data. Indices of muscle oxygenation were averaged across 30 s before the start of the fatiguing protocol and across 10 s at 25, 50, 75, and 100%, respectively. Data analysis for the different conditions (IPC, SHAM, and IPC_tm_) was the same as described for the EMG data.

Baseline values were captured at rest in a seated position. SmO_2_ and total hemoglobin (tHb) were reported as percentage changes from baseline (ΔSmO_2_ and ΔtHb).

Effort and exercise-induced leg muscle pain ratings across the fatiguing protocol were reported for 25, 50, 75, and 100%, respectively (the nearest rating was analyzed). Data analysis for the different conditions (IPC, SHAM, and IPC_tm_) was the same as described for the EMG data.

### Statistical Analysis

All data were screened for normal distribution using the Shapiro–Wilk test. Differences in time-to-exhaustion, state fatigue, ΔMVT, ΔVA, ΔPS100, ΔSS, ΔPS10⋅PS100^–1^ ratio, and ΔM_max_ values were tested using Student’s paired *t*-tests. The effect size Cohen’s *d* was calculated for each paired comparison. Effect sizes of 0.20, 0.50, and 0.80 were considered small, medium, and large, respectively (Cohen, 1988). A two-way (time × condition) repeated measure ANOVA was conducted for each variable recorded during exercise. *Post hoc* tests were performed with Bonferroni adjustments. The effect size was determined by calculating partial eta squared (η_p_^2^). Because only six ‘responders’ to the IPC protocol were identified, interpretation of their results on the basis of *P*-value statistics was not meaningful (du Prel et al., 2009). Therefore, as recommended, interpretation of the responders’ data was based on effect sizes (Cohen’s *d* for the pairwise comparisons and η_p_^2^ for the analyses using the ANOVA) and mean differences with 95% confidence interval [diff. (95%CI)] (Rigby, 1999; Ranstam, 2012; Lakens, 2013; Lee, 2016; Abbott et al., 2018). Cohen’s *d* values ≥ 0.70, representing a medium effect with the tendency to approach a large effect, were considered as meaningful. Partial eta squared values ≥ 0.200 which correspond to a Cohen’s *f* value of 0.50, i.e., a large effect, were considered as relevant (Richardson, 2011). Furthermore, diff. (95%CI) was calculated for the pairwise comparisons and for the ANOVAs (for the latter only if the effect size exceeded the defined threshold).

Data were analyzed using the SPSS statistical package 25.0 (SPSS Inc., Chicago, IL, United States) and statistical significance was accepted at *P* ≤ 0.05. Sample size was calculated with the statistical software package G^∗^Power (version 3.1.4.).

## Results

Six participants were classified as ‘responders’ because they improved their exercise performance following IPC by at least 10% compared to SHAM and completed an IPC_tm_ trial. Therefore, the statistical results for the respective parameter are presented for the whole sample and for the ‘responders’ separately. The interpretation of outcomes of the whole sample is based on the *P*-values, while that of the ‘responders’ is based on the effect size and the diff. (95%CI) (Lakens, 2013). According to that, the order of the statistical parameters is different for the whole sample and the ‘responders.’

### Time-to-Exhaustion Test

#### Whole Sample

**FIGURE 2:**
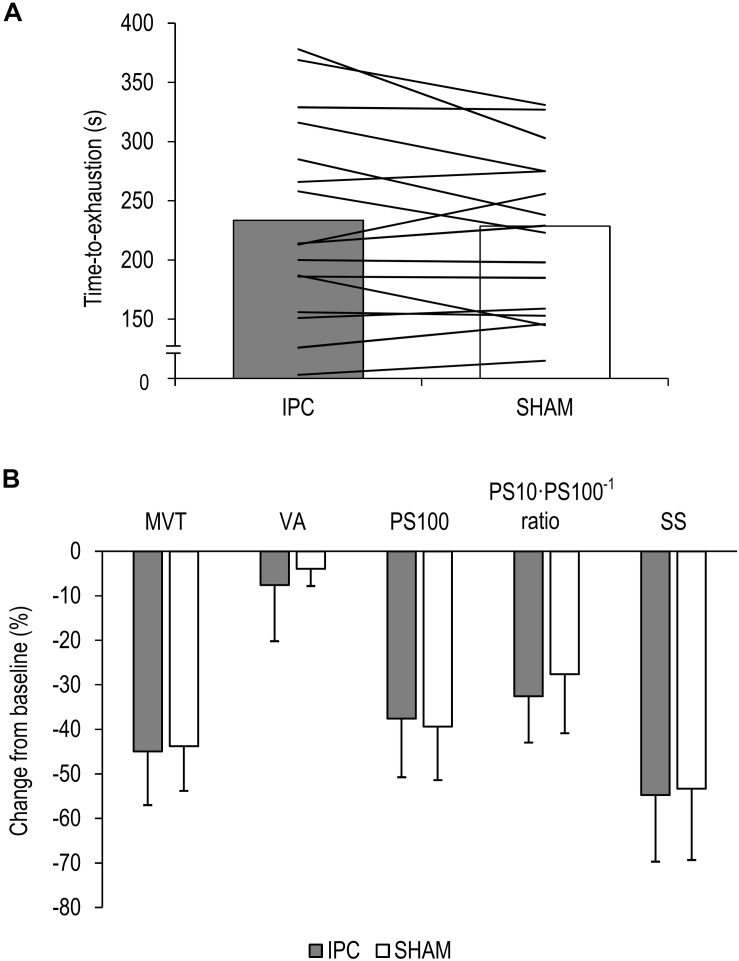
**(A)** Mean values and individual data of all participants for the time-to-exhaustion tests for the ischemic preconditioning (IPC) and sham (SHAM) condition. **(B)** Percentage change from pre-exercise values for all participants for maximal voluntary torque (MVT), voluntary activation (VA), twitch torque in response to paired electrical stimuli (PS100), PS10⋅PS100^–1^ ratio, and twitch torque in response to a single electrical stimulus (SS). Values are presented as mean ± standard deviation.

Time to-exhaustion did not significantly differ between IPC (234 ± 82 s) and SHAM (222 ± 66 s) [*P* = 0.174, diff.: 12 s (−6 to 28 s), *d* = 0.39] (Figure 2A).

#### Responders

**FIGURE 3:**
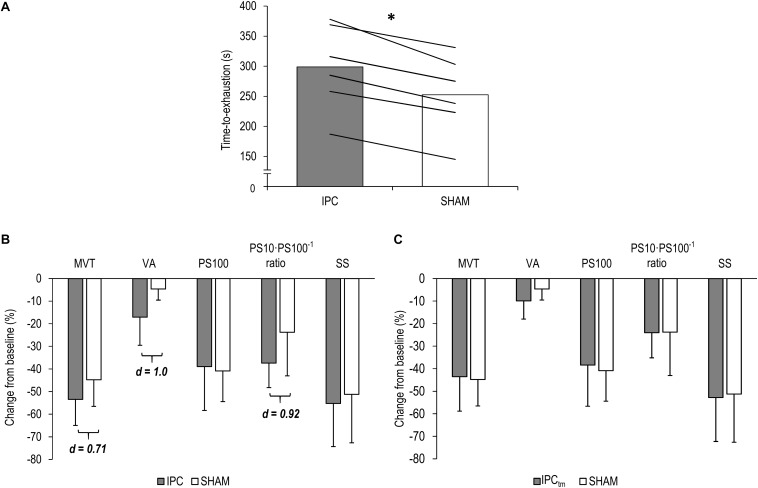
**(A)** ‘Responders’ mean values and individual data for the time-to-exhaustion tests for the ischemic preconditioning (IPC) and sham (SHAM) condition. **(B)** *IPC vs. SHAM* – Percentage change from pre-exercise values of the ‘responders’ for maximal voluntary torque (MVT), voluntary activation (VA), twitch torque in response to paired electrical stimuli (PS100), PS10⋅PS100^–1^ ratio, and twitch torque in response to a single electrical stimulus (SS). **(C)** *Time-matched IPC trial (IPC_tm_) vs. SHAM* – Percentage change from pre-exercise values of the ‘responders’ for MVT, VA, PS100, PS10⋅PS100^–1^ ratio, and SS. Values are presented as mean ± standard deviation. ^∗^*P* < 0.001.

Six participants improved their exercise performance following IPC (299 ± 71 s) by at least 10% compared to SHAM (253 ± 66 s) [*d* = 3.23, diff.: 46 s (31 to 62 s), *P* < 0.001] (Figure 3A) and completed an IPC_tm_ trial. During the IPC_tm_ trial, all ‘responders’ reached the SHAM exercise duration and reported that they were able to continue the submaximal isometric exercise.

### State Fatigue

#### Whole Sample

There were no differences in state fatigue between IPC (12.1 ± 7.5) and SHAM (11.1 ± 6.9) [*P* = 0.247, diff.: 1.0 (−0.7 to 2.6), *d* = 0.34].

#### Responders – IPC vs. SHAM

There were no differences in state fatigue between IPC (14.5 ± 7.5) and SHAM (14.3 ± 6.5) for the six ‘responders’ [*d* = 0.07, diff.: 0.2 (−2.8 to 3.1), *P* = 0.889].

#### Responders – IPC_tm_ vs. SHAM

There were also no differences in state fatigue between IPC_tm_ (14.3 ± 9) and SHAM (14.3 ± 6.5) for the six ‘responders’ [*d* = 0.00, diff.: 0.0 (−5.4 to 5.4), *P* = 1.000].

### Maximal Voluntary Torque

#### Whole Sample

The ΔMVT was not significantly different between IPC and SHAM [*P* = 0.760, diff.: 1.2% (−9.4 to 7.0%), *d* = 0.08]. Percentage changes and absolute values for MVT can be found in Figure 2B and Table 1, respectively.

**TABLE 1:** Neuromuscular function of the quadriceps muscle before and after exercise following ischemic preconditioning (IPC) and the sham intervention (SHAM). Participants who increased their exercise tolerance (‘responders’) after IPC performed a time-matched IPC trial corresponding to the SHAM exercise duration (IPC_tm_).

Parameter	Condition	*Whole sample*		Condition	*Responders*	
				
		Pre	Post		Pre	Post
MVT (N⋅m)						
	IPC	300.764.1	165.950.5	IPC	276.765.7	127.333.9
	SHAM	307.260.0	171.439.5	SHAM	297.361.5	158.015.7
				IPC_tm_	276.754.8	156.858.1
PS100 (N⋅m)						
	IPC	93.312.1	57.510.3	IPC	95.414.9	56.814.8
	SHAM	94.79.2	56.88.9	SHAM	96.611.1	56.19.5
				IPC_tm_	95.015.6	57.815.5
SS (N⋅m)						
	IPC	61.08.9	27.18.3	IPC	61.712.0	26.610.8
	SHAM	63.46.9	29.19.0	SHAM	66.36.7	31.512.0
				IPC_tm_	63.77.3	30.112.3
PS10⋅PS100^–1^ ratio						
	IPC	1.010.08	0.680.11	IPC	1.030.05	0.640.12
	SHAM	1.020.08	0.740.16	SHAM	1.070.03	0.820.22
				IPC_tm_	1.060.02	0.800.13
VM M_max_ (mV)						
	IPC	14.22.2	13.32.9	IPC	14.41.3	13.11.6
	SHAM	14.02.0	13.52.4	SHAM	13.31.8	11.72.0
				IPC_tm_	13.71.9	12.51.6
RF M_max_ (mV)						
	IPC	3.51.6	3.31.3	IPC	3.71.2	3.31.3
	SHAM	4.11.9	3.70.7	SHAM	3.91.0	3.40.4
				IPC_tm_	4.21.4	3.70.9
VL M_max_ (mV)						
	IPC	10.84.4	10.55.3	IPC	9.34.1	7.54.7
	SHAM	9.44.2	8.74.5	SHAM	7.92.8	7.43.1
				IPC_tm_	7.02.7	6.92.6
VA (%)						
	IPC	96.12.7	88.812.0	IPC	96.01.9	79.813.3
	SHAM	95.61.8	91.84.3	SHAM	95.41.8	91.05.2
				IPC_tm_	95.61.5	86.28.4

*Values are expressed as mean ± standard deviation. MVT, maximum voluntary torque; PS100, paired stimuli twitch torque at 100 Hz; SS, single stimulus twitch torque; PS10⋅PS100^–1^ ratio, index of low-frequency fatigue; *M*_max_, maximum *M*-wave; VM, vastus medialis; RF, rectus femoris; VL, vastus lateralis; VA, voluntary activation.*

#### Responders – IPC vs. SHAM

Based on the effect size and mean difference (95%CI), percentage changes in MVT differed between IPC and SHAM [*d* = 0.71, diff.: −8.7% (−35.3 to 17.9%), *P* = 0.416]. Percentage changes and absolute values for MVT can be found in Figure 3B and Table 1, respectively.

#### Responders – IPC_tm_ vs. SHAM

However, ΔMVT was not different between the conditions IPC_tm_ and SHAM [*d* = 0.06, diff.: 1.3% (−26.0 to 28.5%), *P* = 0.905]. Percentage changes and absolute values for MVT can be found in Figure 3C and Table 1, respectively.

### Voluntary Activation

#### Whole Sample

No significant differences between IPC and SHAM were found for ΔVA [*P* = 0.291, diff.:−3.7% (−10.8 to 3.5%), *d* = 0.28]. Relative and absolute values for voluntary activation are presented in Figure 2B and Table 1, respectively.

#### Responders – IPC vs. SHAM

Based on the effect size and mean difference (95%CI), ΔVA differed between IPC and SHAM [*d* = 1.0, diff.:−12.4% (−27.7 to 2.8%), *P* = 0.086]. Relative and absolute values for voluntary activation are presented in Figure 3B and Table 1, respectively.

#### Responders – IPC_tm_ vs. SHAM

The effect size for the percentage changes in ΔVA during IPC_tm_ and SHAM did not reach the defined threshold of *d* = 0.70 [*d* = 0.67, diff.:−5.3% (−14.7 to 4.3%), *P* = 0.202]. Percentage changes and absolute values for VA are presented in Figure 3C and Table 1, respectively.

### Electrically Evoked Twitch Torques

#### Whole Sample

There were no significant differences in ΔPS100 [*P* = 0.409, diff.: 1.8% (−2.8 to 6.4%), *d* = 0.22], ΔSS [*P* = 0.621, diff.: −1.4% (−7.2 to 4.4%), *d* = 0.12], and ΔPS10⋅PS100^–1^ ratio [*P* = 0.100, diff.: −5.0% (−11.1 to 1.1%), *d* = 0.46] between IPC and SHAM. Percentage changes and absolute values for PS100, SS, and PS10⋅PS100^–1^ ratio are presented in Figure 2B and Table 1, respectively.

#### Responders – IPC vs. SHAM

Based on the effect size and mean difference (95%CI), ΔPS10⋅PS100^–1^ ratio differed between IPC and SHAM [*d* = 0.92, diff.:−13.6% (−32.0 to 4.7%), *P* = 0.108]. This difference could not be observed for ΔPS100 [*d* = 0.19, diff.: 1.9% (−10.6 to 14.5%), *P* = 0.694] and ΔSS [*d* = 0.29, diff.:−4.0% (−17.8 to 9.8%), *P* = 0.466]. Percentage changes and absolute values for PS100, SS, and PS10⋅PS100^–1^ ratio are presented in Figure 3B and Table 1, respectively.

#### Responders – IPC_tm_ vs. SHAM

There were no differences between IPC_tm_ and SHAM for ΔPS100 [*d* = 0.45, diff.: 2.5% (−4.4 to 9.4%), *P* = 0.370], ΔSS [*d* = 0.33, diff.:−3.7% (−21.3 to 13.8%), *P* = 0.549], and ΔPS10⋅PS100^–1^ ratio [*d* = 0.02, diff.:−0.25% (−13.9 to 13.4%), *P* = 0.962] of the ‘responders.’ Percentage changes and absolute values for PS100, SS, and PS10⋅PS100^–1^ ratio are presented in Figure 3C and Table 1, respectively.

### Electrically Evoked Potentials

#### Whole Sample

No significant differences in ΔM_max_ between IPC and SHAM were observed for VM, RF, and VL [*P* = 0.415, diff.:−2.8% (−10.0 to 4.4%), *d* = 0.22/*P* = 0.257, diff.: 4.6% (−3.8 to 13.1%), *d* = 0.30/*P* = 0.874, diff.: 1.2% (−15.4 to 17.9%), *d* = 0.04, respectively]. Absolute values for *M*_max_ are presented in Table 1.

#### Responders – IPC vs. SHAM

The effect sizes for the percentage changes of *M*_max_ for VM, RF, and VL recorded during IPC and SHAM did not reach the defined threshold of *d* = 0.70 [*d* = 0.19, diff.: 3.0% (−16.3 to 22.4%), *P* = 0.685/*d* = 0.56, diff.: 10.0% (−12.4 to 32.4%), *P* = 0.283/*d* = 0.67, diff.: − 14.4% (− 41.2 to 12.4%), *P* = 0.211, respectively].

#### Responders – IPC_tm_ vs. SHAM

The same was true for ΔM_max_ of VM, RF, and VL recorded during IPC_tm_ and SHAM [*d* = 0.39, diff.: 3.1% (−6.8 to 13.1%), *P* = 0.429/*d* = 0.03, diff.:−0.5% (−24.1 to 23.1%), *P* = 0.955*/d* = 0.39, diff.: 5.9% (−13.1 to 24.9%), *P* = 0.437, respectively]. Absolute values for *M*_max_ are presented in Table 1.

### EMG Recordings During Exercise

#### Whole Sample

**FIGURE 4:**
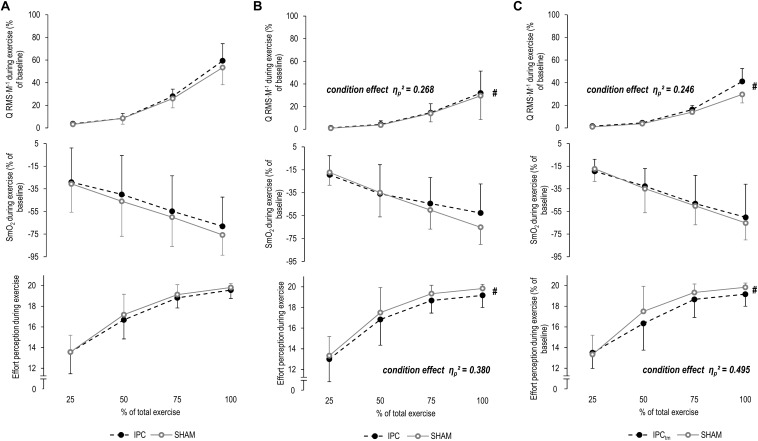
**(A)** Percentage changes in normalized muscle activity of the quadriceps muscle (Q RMS⋅M^–1^), muscle O_2_ saturation of the vastus lateralis (SmO_2_), and effort perception during exercise for the ***whole sample*** for the ischemic preconditioning (IPC) and sham (SHAM) condition. **(B)** *IPC vs. SHAM* – Percentage changes in Q RMS⋅M^–1^, SmO_2_, and effort perception during exercise of the ***‘responders’*** for the IPC and SHAM condition. **(C)** *Time-matched IPC trial (IPC_tm_) vs. SHAM* – Percentage changes in Q RMS⋅M^–1^, SmO_2_, and effort perception during exercise of the ***‘responders’*** for the IPC_tm_ and SHAM condition. Values are presented as mean ± standard deviation.

A time effect was found for ΔQ RMS⋅M^–1^ (*P* < 0.001, *F_4,56_* = 29.495, η_p_^2^ = 0.678, *post hoc* analysis: all *P* ≤ 0.054 compared to the first time point). No condition effect for ΔQ RMS⋅M^–1^ (*P* = 0.497, *F_1,14_* = 0.486, η_p_^2^ = 0.034) or time × condition interaction was observed (*P* = 0.125, *F_4,56_* = 1.892, η_p_^2^ = 0.119) (Figure 4A and Table 2).

**TABLE 2:** Electromyography, near-infrared spectroscopy recordings, and perceptual responses during exercise for the ischemic preconditioning (IPC) and sham condition (SHAM) as well as the time-matched IPC trial corresponding to the SHAM exercise duration (IPC_tm_).

Parameter	Condition	Baseline	Time (% of total exercise duration)			
			
			25	50	75	100

		***Whole sample***				
Q RMS⋅M^–1^						
	*IPC*	0.0380.007	0.0390.007	0.0400.008	0.0450.008	0.0540.012
	*SHAM*	0.0340.006	0.0350.006	0.0360.006	0.0410.005	0.0470.006
SmO_2_ (%)						
	*IPC*	75.49.3	55.126.2	45.528.5	32.924.4	23.521.5
	*SHAM*	75.68.5	51.620.7	39.023.4	28.919.3	17.414.1
THb (g⋅dL^–1^)						
	*IPC*	12.750.31	12.750.36	12.760.39	12.780.45	12.820.44
	*SHAM*	12.790.36	12.800.48	12.840.50	12.870.50	12.920.49
Effort						
	*IPC*	−	13.62.0	16.71.8	18.80.9	19.60.8
	*SHAM*	−	13.61.6	17.21.9	19.10.9	19.80.4
Leg muscle pain						
	*IPC*	−	3.41.6	6.32.1	9.02.2	10.02.3
	*SHAM*	−	3.31.8	6.52.3	8.91.4	10.63.1

		***Responders***				

Q RMS⋅M^–1^						
	*IPC*	0.0370.007	0.0370.007	0.0390.007	0.0420.007	0.0480.005
	*SHAM*	0.0350.004	0.0360.004	0.0370.004	0.0400.003	0.0450.004
	*IPC*_tm_	0.0380.010	0.0390.010	0.0400.009	0.0440.009	0.0510.008
SmO_2_ (%)						
	*IPC*	84.63.8	68.214.4	54.722.7	47.419.9	40.422.0
	*SHAM*	84.25.3	69.59.4	55.620.0	42.515.5	29.913.8
	*IPC*_tm_	79.17.2	63.15.6	53.814.6	41.820.6	32.723.7
THb (g⋅dL^–1^)						
	*IPC*	12.590.33	12.520.39	12.540.47	12.520.54	12.570.55
	*SHAM*	12.620.35	12.510.49	12.570.59	12.620.64	12.660.59
	*IPC*_tm_	12.530.25	12.510.28	12.540.32	12.550.33	12.570.35
Effort						
	*IPC*	−	13.02.0	16.82.3	18.71.1	19.21.1
	*SHAM*	−	13.31.7	17.52.2	19.30.7	19.80.4
	*IPC*_tm_	−	13.51.4	16.32.4	18.71.6	19.21.1
Leg muscle pain						
	*IPC*	−	3.50.8	6.51.9	9.30.5	9.80.4
	*SHAM*	−	3.41.2	6.52.1	9.01.2	9.51.1
	*IPC*_tm_	−	3.20.7	5.82.2	8.31.8	9.21.9

*Values are expressed as mean ± standard deviation. Q RMS⋅M^–1^, averaged root mean square of the electromyography signal normalized to the corresponding maximal M-wave of vastus lateralis, rectus femoris, and vastus medialis; SmO_2_, muscle oxygen saturation; THb, total hemoglobin.*

#### Responders – IPC vs. SHAM

The effect size and mean difference (95%CI) for the main effect condition indicated that muscle activity was slightly higher during IPC compared to SHAM [η_p_^2^ = 0.268, diff.: 0.7% (−0.7 to 2.2%), *F_1,5_* = 1.827, *P* = 0.234]. A time effect was found for ΔQ RMS⋅M^–1^ (η_p_^2^ = 0.691, *F_4,20_* = 11.174, *P* < 0.001, *post hoc* analysis: all *P* ≥ 0.087 compared to the first time point). No time × condition interaction was observed (η_p_^2^ = 0.094, *F_4,20_* = 0.520, *P* = 0.722) (Figure 4B and Table 2).

#### Responders – IPC_tm_ vs. SHAM

The effect size and mean difference (95%CI) for the main effect condition indicated that muscle activity was slightly higher during IPC_tm_ compared to SHAM [η_p_^2^ = 0.246, diff.: 3.0% (−3.0 to 9.0%), *F_1,5_* = 1.630, *P* = 0.258]. A time effect was found for ΔQ RMS⋅M^–1^ (η_p_^2^ = 0.627, *F_4,20_* = 8.408, *P* < 0.001, *post hoc* analysis: all *P* ≥ 0.145 compared to the first time point). The interaction of time × condition showed also a large effect size (η_p_^2^ = 0.273, *F_4,20_* = 1.879, *P* = 0.154) (Figure 4C and Table 2).

### Muscle Oxygenation During Exercise

#### Whole Sample

SmO_2_ decreased (*P* < 0.001, *F_4,60_* = 39.099, η_p_^2^ = 0.723, *post hoc* analysis: all *P* ≤ 0.001 compared to the first time point) and ΔTHb increased (*P* = 0.021, *F_4,60_* = 3.183, η_p_^2^ = 0.197, *post hoc* analysis: all *P* ≥ 0.621 compared to the first time point) over time for both IPC and SHAM. No condition effects for ΔSmO_2_ (*P* = 0.428, *F_1,15_* = 0.663, η_p_^2^ = 0.042) and ΔTHb (*P* = 0.180, *F_1,15_* = 2.012, η_p_^2^ = 0.134) or time × condition interactions for both parameters were observed (*P* = 0.821, *F_4,60_* = 0.382, η_p_^2^ = 0.025/*P* = 0.421, *F_4,60_* = 0.874, η_p_^2^ = 0.063, respectively) (Figure 4A and Table 2).

#### Responders – IPC vs. SHAM

A time effect was found for ΔSmO_2_ (η_p_^2^ = 0.787, *F_4,20_* = 18.475, *P* < 0.001, *post hoc* analysis: all *P* ≤ 0.157 compared to the first time point) but not for ΔTHb (η_p_^2^ = 0.129, *F_4,20_* = 0.591, *P* = 0.674). No condition effects for ΔSmO_2_ (η_p_^2^ = 0.032, *F_1,5_* = 0.164, *P* = 0.703) and ΔTHb (η_p_^2^ = 0.009, *F_1,5_* = 0.038, *P* = 0.855) or time × condition interactions for both parameters were observed (η_p_^2^ = 0.199, *F_4,20_* = 1.243, *P* = 0.325/η_p_^2^ = 0.100, *F_4,20_* = 0.447, *P* = 0.773, respectively) (Figure 4B and Table 2).

#### Responders – IPC_tm_ vs. SHAM

A time effect was found for ΔSmO_2_ (η_p_^2^ = 0.823, *F_4,20_* = 23.210, *P* < 0.001, *post hoc* analysis: all *P* ≤ 0.062 compared to the first time point) but not for ΔTHb (η_p_^2^ = 0.160, *F_4,20_* = 0.760, *P* = 0.566). No condition effects for ΔSmO_2_ (η_p_^2^ = 0.053, *F_1,5_* = 0.281, *P* = 0.619) and ΔTHb (η_p_^2^ = 0.057, *F_1,5_* = 0.240, *P* = 0.650) or time × condition interactions for both parameters were observed (η_p_^2^ = 0.136, *F_4,20_* = 0.786, *P* = 0.548/η_p_^2^ = 0.120, *F_4,20_* = 0.547, *P* = 0.704, respectively) (Figure 4C and Table 2).

### Perception of Effort

#### Whole Sample

There were increases in effort perception over time for both IPC and SHAM (*P* < 0.001, *F_3,45_* = 135.654, η_p_^2^ = 0.900, *post hoc* analysis: all *P* < 0.001 compared to the first time point). No condition effect (*P* = 0.101, *F_1,15_* = 3.046, η_p_^2^ = 0.169) or time × condition interaction was observed for effort perception (*P* = 0.492, *F_3,45_* = 0.541, η_p_^2^ = 0.046) (Figure 4A and Table 2).

#### Responders – IPC vs. SHAM

The effect size and mean difference (95%CI) for the main effect condition indicated that effort perception was lower during IPC compared to SHAM [η_p_^2^ = 0.380, diff.:−0.583 (−1.440 to 0.274), *F_1,5_* = 3.062, *P* = 0.141]. A time effect was found for effort perception (η_p_^2^ = 0.907, *F_3,15_* = 48.519, *P* < 0.001, *post hoc* analysis: all *P* ≤ 0.001 compared to the first time point). No time × condition interaction was observed (η_p_^2^ = 0.048, *F_3,15_* = 0.250, *P* = 0.756) (Figure 4B and Table 2).

#### Responders – IPC_tm_ vs. SHAM

The effect size and mean difference (95%CI) for the main effect condition indicate that effort perception was lower during IPC_tm_ compared to SHAM [η_p_^2^ = 0.495, diff.:−0.583 (−1.261 to 0.094), *F_1,5_* = 4.900, *P* = 0.078]. A time effect was found for effort perception (η_p_^2^ = 0.921, *F_3,15_* = 58.116, *P* < 0.001, *post hoc* analysis: all *P* < 0.001 compared to the first time point). The interaction of time × condition showed also a large effect size (η_p_^2^ = 0.282, *F_3,15_* = 1.964, *P* = 0.203) (Figure 4C and Table 2).

### Perception of Exercise-Induced Leg Muscle Pain

#### Whole Sample

Exercise-induced leg muscle pain perception increased over time during exercise for both IPC and SHAM (*P* < 0.001, *F_3,45_* = 128.337, η_p_^2^ = 0.895, *post hoc* analysis: all *P* < 0.001 compared to the first time point). No condition effect (*P* = 0.541, *F_1,15_* = 0.392, η_p_^2^ = 0.025) or time × condition interaction were found (*P* = 0.519, *F_3,45_* = 0.550, η_p_^2^ = 0.035) (Table 2).

#### Responders – IPC vs. SHAM

A time effect was found for exercise-induced leg muscle pain (η_p_^2^ = 0.916, *F_3,15_* = 54.243, *P* < 0.001, *post hoc* analysis: all *P* ≤ 0.031 compared to the first time point). No condition effect (η_p_^2^ = 0.065, *F_1,5_* = 0.345, *P* = 0.582) or interaction of time × condition (η_p_^2^ = 0.074, *F_3,15_* = 0.399, *P* = 0.677) was observed (Table 2).

#### Responders – IPC_tm_ vs. SHAM

The effect size and mean difference (95%CI) for the main effect condition indicated that exercise-induced leg muscle pain was lower during IPC_tm_ compared to SHAM [η_p_^2^ = 0.562, diff.:−0.479 (−0.966 to 0.008), *F_1,5_* = 6.404, *P* = 0.052]. A time effect was found for exercise-induced leg muscle pain (η_p_^2^ = 0.875, *F_3,15_* = 35.142, *P* < 0.001, *post hoc* analysis: all *P* ≤ 0.083 compared to the first time point). No time × condition interaction was observed (η_p_^2^ = 0.102, *F_3,15_* = 0.571, *P* = 0.565) (Table 2).

## Discussion

The present study was designed to provide further insights into the mechanistic basis for improvements in exercise performance that have been frequently observed after IPC by investigating key-determinants of performance and perceived fatigability. We have not found an improved exercise tolerance for the whole sample during a submaximal isometric voluntary contraction of the knee extensors at 20% MVT following IPC compared to SHAM. This result is in contrast to the finding of Tanaka et al. (2016) who observed an improved time-to-exhaustion of 17.2% during the same task following IPC. The authors have attributed the observed ergogenic effect of IPC to an accelerated muscle deoxygenation response during exercise, which was interpreted as an improved metabolic efficiency. However, our data on central and peripheral determinants of performance fatigability as well as muscle activity, SmO_2_, and perceptual responses during exercise do not support this conclusion. Our results are in accordance with the outcomes of other studies that have neither found an increased exercise performance nor altered physiological and perceptual responses during and after submaximal and maximal exercise following IPC (Tocco et al., 2015; Sabino-Carvalho et al., 2017; Halley et al., 2018, 2019). Explanations for the discrepant outcomes of our study and that of Tanaka et al. (2016) might be that they have not performed a warm-up after the interventions (IPC and the control condition) and before the fatigue protocol. Therefore, the large improvement in time-to-exhaustion of 17.2% following IPC might be in part due to warming-up/priming effects (e.g., increased muscle temperature and improved muscle vascular O_2_ kinetics) induced by the repeated, short-term periods of vascular occlusion with subsequent reperfusion, similar to that induced by prior exercise (Behnke et al., 2002; Burnley et al., 2005). Besides that, the missing SHAM condition and the awareness of the principal investigator regarding the treatment in the experiment of Tanaka et al. (2016) could be additional contributors the discrepant results.

However, it should be not ignored that six participants improved their time-to-exhaustion by more than 10% after IPC compared to SHAM. Although the subsample analysis of six participants should be interpreted with caution, the time-matched comparisons (IPC_tm_ vs. SHAM) suggest that performance fatigability and its central and peripheral determinants were not affected by IPC. Furthermore, SmO_2_ data were also similar between all conditions. These data do not support the assertion that IPC improves metabolic efficiency and/or blood flow in the active skeletal muscles during exercise, which are thought to be the main mechanisms for the ergogenic effect of IPC (Incognito et al., 2016). Based on the effect sizes, it could be speculated that the longer time-to-exhaustion of the ‘responders’ following IPC was associated with greater impairments in neuromuscular function as indicated by a larger decrease in MVT, voluntary activation, and low-frequency twitch torque. Furthermore, data indicate that effort perception was lower and muscle activity was slightly higher during both IPC and IPC_tm_ compared to SHAM, suggesting a reliable impact of IPC on effort perception and muscle activity during submaximal isometric exercise.

An improved exercise performance and a lower effort perception during exercise in response to IPC has also been shown by others (Cruz et al., 2015; Paradis-Deschenes et al., 2018). In line with our data for the ‘responders,’ Cruz et al. (2015) have found that the increased constant-load cycling performance after IPC was accompanied by a lower effort perception and higher muscle activity. There is a general finding that interventions which can reduce effort perception have the potential to increase endurance performance (McCormick et al., 2015). As a key-determinant of endurance performance, effort perception is thought to be involved in processes related to self-regulation, exercise behavior, and task disengagement (Marcora, 2008; Venhorst et al., 2018). Therefore, a lower effort perception following IPC might have enabled the participants to continue the submaximal isometric task for longer and allowed them to tolerate larger impairments in neuromuscular function. Although there is still controversy about whether effort perception results from a centrally mediated feedforward mechanisms (i.e., corollary discharge model) and/or afferent feedback from the working and respiratory muscles (i.e., afferent feedback or combined model), it is well accepted that neural processing of sensory signals in the brain is involved (Marcora, 2009; Pageaux, 2016). Since peripheral factors of performance fatigability (i.e., contractile function) and SmO_2_ data were not altered by IPC at the same point in time, processes within the nervous system might have played a role in the reduced effort perception following IPC in the ‘responders’. Recently, it has been speculated that IPC might desensitize small-diameter group III and IV muscle afferents leading to less inhibition at the supraspinal and/or spinal level during exercise. The authors proposed that these mechanisms might be responsible for the lower effort perception and higher muscle activity observed during constant-load cycling following IPC (Cruz et al., 2015, 2017). This assumption has been criticized for several reasons, e.g., the definition of effort perception in the experiment of Cruz et al. (2015) and the importance of small-diameter muscle afferents not only for inhibitory processes in the central nervous system but also for the upregulation of cardiovascular and ventilatory function during exercise (see Commentaries on Viewpoint of Cruz et al., 2017: Could small-diameter muscle afferents be responsible for the ergogenic effect of limb ischemic preconditioning?). An alternative explanation for the lower effort perception during exercise might be that periods of local ischemia increase the excitability of the corticospinal pathway at rest. McNulty et al. (2002) investigated the effect of 40 min of local ischemia on motor potentials of the first dorsal interosseous muscle evoked by transcranial magnetic stimulation. The authors have found that the motor-evoked potentials decreased progressively during the ischemic period but were significantly elevated for up to 20 min after the restoration of blood flow, which coincided with heightened afferent neural volleys. If the excitability of neurons at the supraspinal and/or spinal level of the respective muscle is increased, it might need to receive less input to generate the same muscle activation signal. In this case, a lower effort perception for a given torque output should be expected. However, currently it is not known if IPC increases corticospinal excitability at rest and/or during submaximal voluntary contractions and how long this effect persists. Due to the transient nature of these potential changes, only endurance tasks performed directly after the treatment might benefit from these acute neural adjustments.

Exercise-induced muscle pain perception of the ‘responders’ was not different between IPC and SHAM, but seemed to be lower during IPC_tm_ compared to SHAM. Because the IPC_tm_ trial was the last one for each ‘responder’, it might be that the repetitive application of IPC has altered pain perception and/or tolerance due to habituation to the noxious stimuli. This phenomenon was previously observed in response to high-intensity training (O’Leary et al., 2017).

## Conclusion

IPC did neither affect exercise tolerance, performance fatigability, as well as its central and peripheral determinants, nor muscle activity, SmO_2_, and perceptual responses during submaximal exercise. However, it should be not ignored that six out of 16 participants improved their time-to-exhaustion during the submaximal isometric endurance task following IPC. Our data suggest that this subsample of ‘responders’ was able to endure for longer and tolerated greater exercise-induced impairments in neuromuscular function after IPC. Interestingly, IPC seemed to be associated with a lower perception of effort during exercise. Since effort perception is considered as a key-determinant of endurance performance, a lower effort perception might have contributed to the improved time-to-exhaustion of the ‘responders.’ Future studies should aim to identify the underlying factors that contribute to inter-individual differences in the responsiveness to IPC, so that IPC can effectively be used as an ergogenic aid. The present findings support the assumption that there are ‘responders’ and ‘non-responders’ to IPC, which might contribute to the heterogeneous findings regarding the ergogenic effect of IPC on exercise performance.

### Limitations

Although conditions were randomized, pre-exercise MVT values of the ‘responders’ were higher in the SHAM condition compared to IPC as well as compared to IPC_tm_, which resulted in a slightly higher absolute load during the 20% MVT fatigue protocol in the SHAM condition. During SHAM, the ‘responders’ generated 55.9 ± 17.1 N⋅m (20.2 ± 0.6% MVT) compared to 53.0 ± 14.1 N⋅m (20.2 ± 0.5% MVT) in the IPC condition [*d* = 0.59, diff.:−2.9 N⋅m (−7.9 to 2.0 N⋅m), *P* = 0.191] and 54.1 ± 14.5 N⋅m (20.7 ± 0.8% MVT) in the IPC_tm_ condition [*d* = 0.52, diff.:−1.8 N⋅m (−5.8 to 2.1 N⋅m), *P* = 0.283]. However, muscle activity was slightly higher during both IPC conditions compared to SHAM and contractile function was not different between the time-matched conditions (IPC_tm_ vs. SHAM). Hence, the slightly higher absolute load during SHAM was probably not a significant contributor to the observed results of the ‘responders’. Data of the remaining participants corroborate this assumption, because for six out of 10 subjects time-to-exhaustion was longer or similar despite higher pre-exercise MVT values, irrespective of the condition. Since our participants were young, healthy, and active males, conclusions for other populations should be drawn with caution. In addition, SmO_2_ data were only captured for the VL and we cannot rule out that muscle oxygenation was different in the other muscles of the quadriceps after IPC.

## Data Availability Statement

The datasets generated for this study are available on request to the corresponding author.

## Ethics Statement

The studies involving human participants were reviewed and approved by Ethics Committee University of Rostock, St.-Georg-Str. 108, 18055 Rostock. The patients/participants provided their written informed consent to participate in this study.

## Author Contributions

MB and FH designed the study, collected, analyzed, and interpreted the data, and wrote the manuscript. SB, TM, and VZ contributed to writing, reviewing, and editing of the manuscript. All authors approved the final version of the manuscript.

## Conflict of Interest

The authors declare that the research was conducted in the absence of any commercial or financial relationships that could be construed as a potential conflict of interest.
